# Food security status of patients with type 2 diabetes and their adherence to dietary counselling from selected hospitals in Addis Ababa, Ethiopia: A cross-sectional study

**DOI:** 10.1371/journal.pone.0265523

**Published:** 2022-04-14

**Authors:** Robel Tezera, Zekariyas Sahile, Delelegn Yilma, Equilnet Misganaw, Endale Amare, Jemal Haidar

**Affiliations:** 1 Department of Medical Radiological Technology, Division of Public Health, School of Medicine, Addis Ababa University, Addis Ababa, Ethiopia; 2 Department of Public Health, College of Medicine and Health Science, Ambo University, Ambo, Ethiopia; 3 CIHLMU Center for International Health, University Hospital, LMU, Munich, Germany; 4 Food Science and Nutrition Research Directorate, Ethiopian Public Health Institute, Addis Ababa, Ethiopia; 5 School of Public Health, College of Health Science, Addis Ababa University, Addis Ababa, Ethiopia; Quensland University of Technology, AUSTRALIA

## Abstract

**Background:**

Even though adherence to dietary counselling for patients with diabetes is essential for improving health and preventing complications, access to an adequate and quality diet is challenging for patients living in a food-insecure household. The availability of data in this regard is limited in Ethiopia. Thus, this study assessed the food security status of patients with type 2 diabetes, their adherence to dietary counselling, and contributing factors at public hospitals in Addis Ababa, Ethiopia.

**Methods:**

This was a facility-based cross-sectional study among 602 patients with Type 2 diabetes in Addis Ababa, Ethiopia, from July to August 2019. Patients were selected randomly after the total number of samples was proportionally allocated to four public hospitals. Relevant information was collected by trained data collectors using a pre-tested questionnaire. Data were entered into Epi-info version 7 and exported to SPSS version 24 for data analysis. Logistic regression analysis was employed to identify factors associated with adherence to dietary counselling.

**Result:**

The proportion of nonadherence to dietary counselling among patients with type 2 diabetes was 67.3% (95%CI: 63.5%-71.1%). Nearly half (50.7%) of the respondents were food insecure. Of these, mildly food insecure, moderately food insecure, and severely food insecure were 8.5%, 29.2%, and 13%, respectively. Physical activity (AOR = 1.7; 95%CI: 1.1–2.9); diabetes knowledge (AOR = 1.8; 95%CI: 1.2–2.6); lack of access to information (AOR = 1.6; 95%CI: 1.1–2.6); moderately food insecure (AOR = 2.2; 95%CI: 1.3–3.7); and severely food insecure (AOR = 5.6; 95%CI: 2.1–15.0) were the major significant factors associated with nonadherence to dietary counselling.

**Conclusion:**

Over two-thirds of patients with diabetes did not adhere to dietary counselling, which appears high. As a result, improving diabetes education, information access, and food security status should be considered to ensure dietary counselling adherence among type 2 diabetes patients.

## Introduction

Type 2 diabetes mellitus (T2DM) has become a significant public health problem around the globe. The World Health Organization identified diabetes as one of the four priority Non-Communicable diseases (NCDs) along with cardiovascular disease, cancer, and chronic respiratory disease [1]. Globally, there were more than 500 million estimated type 2 diabetes cases in 2018 [2], and 4 million people died because of diabetes in 2017 [3]. If countries do not implement appropriate action, the number of people with type 2 diabetes is expected to reach 552 million by 2030 [4].

In Ethiopia, T2DM is a significant public health problem, with an estimated prevalence of 3.2% in 2015 [5]. In 2017, the projected prevalence of T2DM was 7.5% (% population ages 20–79), and approximately 30,000 deaths attributed to it [3]. This was higher than the estimated prevalence in Uganda (2.5%), Rwanda (4.3%), and Kenya (2.9%) [3]. Diabetes-related death is often associated with complications such as hypertension [6], neuropathy, retinopathy [7], cardiovascular disease [8], and foot ulcer [9]. A study conducted on patients with diabetes admitted in large referral hospitals in Addis Ababa reported that 33% of the patients were admitted with a diabetes foot ulcer, about 20% with diabetes cardiovascular disease/stroke, and 10% with renal failure [10]. While lifestyle and dietary counselling in-hospital care are generally considered adequate by patients, poor patient compliance with dietary counselling is a major challenge in diabetes management [11], leading to several other complications and hospitalization [12, 13].

Managing T2DM requires an integrated and holistic approach where a healthy lifestyle and dietary counselling play essential roles in managing diabetes. Badedi et al. (2016) reported that diabetes who complied with the recommended diabetes diet had lower HbA1c [14]. Self-management support and Diabetic Self-management education on dietary practice significantly impacted managing complications in diabetes [14–17].

The reason for nonadherence to dietary counselling could be affected by lack of information, quality of dietary counselling, poor health-seeking behaviors, inadequate knowledge, restriction of culturally sensitive foods, and lack of family support [18, 19]. On the other hand, food insecurity is also a significant challenge for patients with diabetes to adhere to recommended dietary guidelines [20]. The 2016 Global Hunger Index score for Ethiopia set by the International Food Policy Research Institute was 33.4, a slight decline from the previous scoring of 33.9 [21]. The estimated prevalence of severe food insecurity in Ethiopia was 19.1% in 2016–2018 [22] though the disaggregate evidence with T2DM in Ethiopia is not elucidated.

Available evidence from different literature shows significant nonadherence rates by patients with T2DM to dietary recommendations [23, 24]. Even though adherence to dietary counselling for patients with diabetes is essential for improving health and preventing complications, access to an adequate and quality diet is challenging for patients living in a food-insecure household. The availability of data in this regard is limited in Ethiopia. Thus, this study assessed the food security status of patients with type 2 diabetes, their adherence to dietary counselling, and contributing factors at public hospitals in Addis Ababa, Ethiopia.

## Methods

### Study design, settings, and period

A facility-based cross-sectional study was conducted among adult patients with T2DM at selected public hospitals in Addis Ababa, Ethiopia, from July 10 to August 10, 2019. Addis Ababa city administration has 11 public hospitals; we used simple random sampling techniques to select four.

### Inclusion and exclusion criteria

All patients with T2DM aged 18 and above who had a regular monthly medical check-up at the selected public hospitals, at least one year on diabetes treatment, and could speak in Amharic or English were eligible for this study. Patients with mental disorders, gestational diabetes, and admitted as an inpatient were excluded.

### Sample size determination and sampling techniques

The sample size was determined using the sample size determination formula for a single population proportion

$$n=\frac{{\left(Z_{\frac{\alpha }{2}}\right)}^{2}*p\left(1-p\right)}{d^{2}}$$

Z _α/2_ is the standard normal distribution at 95% confidence intervals (1.96),

p is prevalence (51.4%) taken from a previous study [25]

d is the margin of error set at 0.05

$$n=\frac{1.96^{2}*0.514\left(1-0.514\right)}{0.05^{2}}$$

n=384

We multiplied the sample size by design effect (1.5*384) to maximize its representation for other public hospitals in Addis Ababa, Ethiopia. After considering 10% for the non-response rate, the total sample size was 634.

Sampling frames were constructed from monthly regular medical check-up chart registration for the selected four hospitals from July 10 to August 10, 2019. The total population size built from the selected hospitals was 760, 1373, 1537, and 1630, comprising 5,300 eligible participants. The sample size was then allocated based on the probability proportional to size (PPS) of each hospital population; large samples were allocated for hospitals with large amounts of patients. Consequently, study participants were selected independently from each hospital using simple random sampling techniques and contacted during their monthly medical check-ups.

### Data collection tools and methods

Data was collected using a pre-tested interview administered questionnaire. The questionnaire comprised of demographic data, mode of diagnosis, family history of diabetes, physical exercise, cigarette smoking status, alcohol drinking habit, family support, check fasting blood glucose, access to nutritional education, and access to information, perceived barrier, a perceived dietary adherence questionnaire (PDAQ) [26], Food Insecurity Experience Scale (FIES) [27, 28], and a Revised Michigan Diabetes Knowledge Scale [29].

A PDAQ was used to measure patients’ dietary counseling adherence [26]. The PDAQ scale consists of eight questions structured to dietary guidelines for patients with diabetes, including healthy diet planning, recommended fruits and vegetable servings, consumption of low glycemic index carbohydrate-containing foods, high sugar foods, and high fiber, healthy omega-3 oils, and high-fat foods. One item addresses appropriate carbohydrate spacing. We customized the tool to local dietary recommendations for patients with T2DM by providing examples for each item from locally available evidence [30–33]. The PDAQ is a 7-point scale, and participants were asked about their perceptions on diet consumption with an answer to the question phrased as "On how many of the last 7 days did you…?" explained with local diet examples. The scores ranged from lowest 0 to highest 7; the total score was computed by summation of items, and the total summed range of scores was from 0–56. The higher PDQA score represents more adherence to dietary counselling. The PDAQ score ≥ 28 is considered adherent during analysis, and < 28 is considered nonadherent.

Patients’ food insecurity experience was determined using the FIES/Food and Agriculture Organization tool composed of 8 items with 1 = yes and 0 = no responses [27, 28]. Revised Michigan Diabetes Knowledge Scale assessed patient level of T2DM related knowledge with 18 items; the total score was converted to 100% [29]. The perceived barrier to dietary adherence was measured by 5 items scale focused on the barrier to dietary behavior taken from the barriers to self-care scale [34]. The rating is a 4-point Likert scale, and the total summed range of scores was from 4–20. Perceived barrier summed score ≥ 50% was labeled as “Yes,” and score < 50% was labeled as “No” [34, 35].

Data were collected by four Nurse professionals recruited from each hospital, and training was provided for the data collectors on the tool. The data collectors completed the tool by interviewing all study participants. Additionally, we collected fasting blood glucose level (FBG), complications related to diabetes, comorbidities, and disease duration by reviewing patient medical records using a pre-structured checklist.

A pre-test was conducted to assess the questionnaire’s clarity, length, completeness, and reliability. Internal validity was maintained by adjusting confounding variables and adopting a well-tested instrument for data collection. Additionally, factorial analysis using the model-fit measures was used to assess the model’s overall goodness of fit.

### Data analysis

The collected data were checked for completeness, entered to Epi-info version 7, and transported to SPSS version 24 for data analysis. Descriptive statistics such as frequencies, percentages, measures of central tendencies, and dispersions were used to explain the study subjects’ selected characteristics. Variance inflation factors (VIF) were used to test Multicollinearity between independent variables. Bivariate analysis was used to determine the association between independent variables and dietary counseling adherence. Independent variables with a p-value of 0.2 and less during the bivariate test were included in the multivariable logistic regression model. Independent samples t-test was employed to compare the mean perceived adherence to dietary counselling score difference among food secure and insecure groups. Logistic regression analyses were carried out to compare independent predictors of dietary Counselling adherence between food secure and insecure groups. A 5% significance level was used for the inferential statistics to guide statistical significance with 95%CI of the crude and adjusted odds ratio. The final model fitness was tested using Omnibus Tests of Model Coefficients and Hosmer and Lemeshow Test.

### Ethical consideration

We obtained Ethical clearance from Addis Ababa Public Health Research and Emergency Management Directorate. Written informed consent was sought from all selected participants after the study’s nature was communicated to all participants in their local language. Participants’ right to withdraw from the survey was respected, and confidentiality was maintained during the interview.

### Operational definitions

#### Perceived adherence to dietary counselling

If dietary adherence score is ≥ 28, considered as adherent; if dietary adherence score is < 28, considered as nonadherent [36].

#### Food security status

If the FIES raw score is 0 considered as food-secure; if the raw score is 7 and above considered as severely food-insecure; if the raw score is between 3 and 6 considered as moderately food-insecure; if the raw score is either 1 or 2 considered as mildly food-insecure [27, 28].

#### T2DM-related knowledge

If participants correctly answered 60% and above the Michigan Diabetes knowledge scale, they were categorized as adequate knowledge; the knowledge score is below 60%, categorized as inadequate knowledge [29].

#### Glycemic control

If the fasting blood glucose is between 80–130 mg/dl considered as controlled glycemic level; if fasting blood glucose is either less than 80mg/dl or greater than 130mg/dl considered as a poorly controlled glycemic level [37].

#### Physical exercise

If participants are not engaged in physical exercise labeled as "No"; if the participants engaged in physical exercise (at least one day/week) labeled as "Yes". [38].

#### Access for information

Participants have access to information if they have access at least from one source, including journals, mass media, social networks, leaflets, social groups, etc. [39].

#### Perceived barrier to dietary adherence

If the participant’s score is 50% and above labeled as “yes” for the perceived barrier, and if the total participant score is below 50% labeled as “No” for perceived barrier [34, 35].

## Results

### Sociodemographic characteristics

Of the 634 eligible patients with T2DM, 602 (96%) participated in this study. Of these, 288 (47.8%) were males and 314 (52.2%) females. The mean age (±SD) of respondents was 52.3 (±13.7). Over half, 326 (54.2%) were married, 124 (20.6%) had no formal education, and 200 (33.2%) participants were employed (Table 1).

**Table 1:** Sociodemographic characteristics of patients with type 2 diabetes who had a regular medical check-up at selected public hospitals in Addis Ababa, 2019.

Characteristics		Frequency	Percentage (%)
Mean age (±SD)		52.3(±13.7)	
Sex	Male	288	47.8
	Female	314	52.2
Marital Status	Single	129	21.4
	Married	326	54.2
	Divorced	40	6.6
	Separated	43	7.1
	Widowed	64	10.6
Educational Status	No formal education	87	14.5
	Primary	124	20.6
	Secondary	199	33.1
	Tertiary	192	31.9
Employment Status	Unemployed	67	11.1
	Employed	200	33.2
	Pensioners	76	12.6
	Housewives	120	19.9
	Private Business	139	23.1

### Clinical, food security, and other related characteristics

Out of 602 respondents, 234 (38.9%) were diagnosed with diabetes incidentally and 193 (32.1%) by screening. Over half the respondents, 342 (56.8%), had no family history of diabetes. About one-third, 208 (34.6%), developed diabetes-related complications. One-third of the respondents, 203 (33.7%), never engaged in physical activity, and only 115 (19.1%) engaged in physical activity daily. The majority, 526 (87.4%) and 435 (72.3%) were non-smokers and had no drinking habits, respectively. Over one-third, 224 (37.2%), were diagnosed with non-communicable diseases other than diabetes.

Based on the blood glucose results, 439 (72.9%) of patients with T2DM had poorly controlled FBG, and 307 (51%) reported a disease duration of more than five years. Only 50 (8.3%) checked their fasting blood glucose daily, and 467 (77.6%) had family support regarding their disease. More than half of the respondents, 312 (51.8%), got nutritional education from the hospital, and 322 (53.5%) had access to information related to diabetes nutrition. About two-thirds, 394 (65%), perceived barriers to dietary adherence, and 352 (58.5%) had inadequate knowledge of diabetes.

More than half of the respondents, 305 (50.7%), were food insecure. The proportion of mildly food insecure, moderately food insecure, and severely food insecure were 51 (8.5%), 176 (29.2%), and 78 (13%), respectively (Table 2).

**Table 2:** Clinical and other related characteristics of patients with T2DM who had regular medical check-ups at selected public hospitals in Addis Ababa, 2019.

Items	Response	Frequency	%
Mode of diagnosis	Incidental	234	38.9
	At screening	175	29.1
	Symptomatic	193	32.1
Family History of diabetes	Yes	260	43.2
	No	342	56.8
Comorbidities	Yes	224	37.2
	No	378	62.8
How often did you engage in physical exercise?	Never	203	33.7
	2 days per week	146	24.3
	3 days per week	97	16.1
	4 days per week	41	6.8
	Daily	115	19.1
Cigarette Smokers	Yes	76	12.6
	No	526	87.4
Alcohol Drinking Habit	Yes	167	27.7
	No	435	72.3
Family Support	Yes	467	77.6
	No	135	22.4
T2DM related complication	Yes	208	34.6
	No	394	65.4
Disease (T2DM) duration	<5 years	295	49
	> = 5 years	307	51
Glycemic Control	Controlled	163	27.1
	Poorly controlled	439	72.9
Check fasting blood glucose level every day	Yes	50	8.3
	No	552	91.7
Get nutritional education from the hospital	Yes	312	51.8
	No	290	48.2
Access to information related to diabetes nutrition	Yes	322	53.5
	No	280	46.5
A perceived barrier to dietary adherence	Yes	208	34.6
	No	394	65.4
Diabetes-related Knowledge	Adequate Knowledge	250	41.5
	Inadequate Knowledge	352	58.5
Food Security	Food Secure	297	49.3
	Mild Food Insecurity	51	8.5
	Moderate Food Insecurity	176	29.2
	Severe Food Insecurity	78	13

### Adherence to dietary counselling

About two-thirds, 67.3% (95%CI: 63.5%-71.1%), of patients with T2DM were nonadherent to dietary counselling. The full PDAQ distribution of patients with T2DM is presented in the S1 Table.

### Food security status and adherence to dietary counselling

The mean score of individual PDQA items was compared between food-secure and food-insecure patients. The mean score of healthy eating plan, fruit, and vegetables, carbohydrate-containing foods with a low-glycemic index, and omega-3 oils were higher among food-secure groups (p<0.05). The mean score of carbohydrate spacing throughout the day was also higher among food-secure patients (p<0.001). On the contrary, food-secure patients consume more fatty foods than food-insecure patients (p<0.001) (Table 3).

**Table 3:** Independent samples t-test for mean adherence to dietary counselling score of the food secure and food insecure patients with T2DM.

PDAQ Items	Food Secure (n = 297) Mean (±SD) days/week	Food Insecure (n = 305) Mean (±SD) days/week	Sig (P-value)
Follow Healthy eating plan	4.65 (±2.3)	3.46 (±2.3)	<0.001*
Consume Fruit and vegetable**	3.32 (±2.1)	1.92 (±1.6)	0.001*
Consume Carbohydrate-containing foods with a low Glycemic Index	2.27 (±1.7)	1.9 (±1.7)	0.007*
Avoid Foods high in sugar	6.18 (±1.1)	6.26 (±1.2)	0.411
Consume Foods high in fiber**	1.49 (±1.4)	1.55 (±1.6)	0.615
Spaced carbohydrates evenly throughout the day**	1.43 (±1.3)	0.91 (±1.2)	<0.001*
Consume Foods that contained omega-3 oils**	1.24 (±1.4)	0.73 (±1.1)	<0.001*
Avoids Foods high in fat**	5.28 (±1.5)	5.73 (±1.3)	<0.001*

** The equal variance is not assumed (Sig. value for Levene’s test is less than 0.05).

### Factors affecting nonadherence to dietary counselling

Multivariate logistic regression was used to assess the contributing factors for nonadherence to dietary counselling. The logistic regression model was statistically significant, p<0.05. The model explained 20% (Nagelkerke R^2^) of the variance in dietary nonadherence and correctly classified 70.8% of cases. As shown in Table 3, only four independent variables made a unique, statistically significant contribution to the model (physical activity, diabetes knowledge, food security, and access to information related to diabetes nutrition).

The strongest predictor of nonadherence to dietary counselling, recording an odds ratio of 5.6, is food insecurity. The result shows that severely food insecure patients had more than fivefold higher odds of being nonadherent to dietary counselling than food-secure counterparts (AOR = 5.6; 95%CI: 2.1–15.0). Similarly, moderately food insecure patients had more than twofold higher odds of being nonadherent to dietary counselling than food-secure patients (AOR = 2.2; 95%CI: 1.3–3.7). Physical activity (AOR = 1.7; 95%CI: 1.1–2.9); diabetes knowledge (AOR = 1.8; 95%CI: 1.2–2.6); and access to information related to diabetes nutrition (AOR = 1.6; 95%CI: 1.1–2.6) were also significantly associated with nonadherence to dietary counselling of patients with T2DM (Table 4).

**Table 4:** Factors associated with nonadherence to dietary counselling of patients with T2DM who had regular medical check-ups at selected public hospitals in Addis Ababa, 2019.

Variable	Responses	Adherent Number (%)	Non-adherent Number (%)	COR (95%CI)	AOR (95%CI)
Age				1.0 (0.9–1)	
Sex	Male	93 (32.6)	195 (67.7)	1	
	Female	104 (33.1)	210 (66.9)	0.9 (0.7–1.4)	
Marital Status	Single	43 (33.3)	86 (66.7)	1	
	Married	105 (32.2)	221 (67.8)	1.1 (0.7–1.6)	
	Divorced	11 (27.5)	29 (72.5)	1.2 (0.6–2.8)	
	Separated	20 (46.5)	23 (53.5)	0.5 (0.2–1.2)	
	Widowed	18 (28.1)	46 (71.9)	1.3 (0.7–2.5)	
Education	Illiterate	13 (14.9)	74 (85.1)	3.1 (1.7–5.8)**	1.6 (0.8–3.1)
	Literate	184 (35.)	331 (64.3)	1	1
Occupation	Employed	83 (41.5)	117 (58.5)	1	1
	Unemployed	114 (28.4)	288 (71.6)	1.8 (1.3–2.6)**	1.2 (0.8–1.9)
Engaged in Physical Exercise	Yes	162 (40.6)	237 (59.4)	1	1
	No	35 (17.2)	168 (82.8)	3.3 (2.1–4.9)**	1.7 (1.1–2.9)*
Cigarette Smoker	Yes	173 (33.9)	353 (67.1)	1.1 (0.6–1.8)	
	No	24 (31.6)	52 (68.4)	1	
Alcohol Drinking Habit	Yes	51 (30.5)	116 (69.5)	1.2 (0.8–1.7)	
	No	146 (33.6)	289 (66.4)	1	
Family History of Diabetes	Yes	85 (32.7)	175 (67.3)	1	
	No	112 (32.7)	230 (67.3)	0.9 (0.7–1.4)	
Comorbidities	Yes	76 (33.9)	148 (66.1)	0.9 (0.6–1.3)	
	No	121 (32)	76 (68)	1	
Family Support	Yes	168 (36)	299 (64)	1	1
	No	29 (21.5)	106 (78.5)	2.1 (1.3–3.2)**	1.6 (0.9–2.6)
T2DM related complication	Yes	55 (26.4)	153 (73.6)	1.6 (1.1–2.3)*	1.2 (0.8–1.8)
	No	142 (36)	252 (64)	1	
Check blood glucose	Regularly check BG	181 (32.8)	371 (67.2)	1	
	Don’t check BG	16 (32)	34 (68)	1.1(0.6–1.9)	
Access to nutritional education	Yes	118 (37.8)	194 (62.2)	1	1
	No	79 (27.2)	211 (72.8)	1.6 (1.2–2.3)**	1.7 (0.8–1.9)
Access to information	Yes	139 (43.2)	183 (56.8)	1	1
	No	58 (20.7)	222 (79.3)	2.9 (2.0–4.2)**	1.6 (1.1–2.6)*
Disease Duration	< 5 years	84 (27.4)	223 (72.6)	1	1
	> = 5 years	113(38.3)	182 (61.7)	1.7 (1.2–2.3)**	1.2 (0.8–1.7)
Glycemic Control	Controlled	61 (37.4)	102 (62.6)	1	
	Poorly controlled	136 (31)	303 (69)	1.3 (0.9–1.9)	
Food Security status	Secure	131(44.1)	166 (55.9)	1	1
	Mildly Insecure	19 (37.3)	32 (62.7)	1.3 (0.7–2.4)	1.1 (0.5.0–2.1)
	Moderately insecure	41 (23.3)	135 (76.7)	2.6 (1.7–3.9)**	2.2 (1.3–3.7)**
	Severely Insecure	6 (7.7)	72 (92.3)	9.5 (3.9–22.4)**	5.6 (2.1–15.0)**
Perceived barrier to Dietary adherence	Yes	56 (26.9)	152 (73.1)	1.5 (1.1–2.2) *	1.7 (0.5–1.3)
	No	141 (35.8)	253 (64.2)	1	1
T2DM related Knowledge	Adequate	115 (46)	135 (54)	1	1
	Inadequate	82 (23.3)	270 (76.7)	2.8 (2.0–4.0)**	1.8 (1.2–2.6)**

Note. Each adjusted odds ratio is adjusted for the remaining variables shown in the Table. AOR = adjusted odds ratio; COR = crude odds ratio.

*P < 0.05

**P < 0.01.

We calculated food security effect modification’s significance using multiple logistics regression models, including interaction terms between food security and dietary nonadherence predictors (access to information, physical exercise, and T2DM related knowledge). The regression analysis revealed a significant interaction between food security and access to information (p<0.001); and between food security and physical exercise (p = 0.008). The interaction effect between food security and T2DM related knowledge was not significant (p = 0.829) (S2 Table).

Thus, we computed the stratum-specific odds ratio based on food security status for all independent variables. In the stratum-specific analysis, physical exercise (AOR = 2.8; p- = 0.007) and diabetes knowledge (AOR = 1.8; p = 0.034) were significantly associated with nonadherence to dietary counselling among food-secure patients. In food-insecure patients, occupation (AOR = 2.0; p = 0.04), information access (AOR = 2.8; p = 0.002), and diabetes knowledge (AOR = 1.7; p = 0.03) were significantly associated with non-adherence to dietary counselling (Table 5).

**Table 5:** Stratum-specific multiple regression analysis of dietary nonadherence predictors based on the food security status of patients with T2DM at selected public hospitals in Addis Ababa, 2019.

Variable	Responses	Food Secure		Food-Insecure	
		COR (95%CI)	AOR (95%CI)	COR (95%CI)	AOR (95%CI)
Age (Mean)		0.9 (1.0–1.1)		1.0 (0.9–1.0)	
Sex	Male	1		1	
	Female	0.9 (0.6–1.4)		0.9 (0.6–1.7)	
Education	Illiterate	1.6 (0.5–4.8)		2.7 (1.2–5.9) *	1.6 (0.7–3.8)
	Literate	1		1	1
Occupation	Employed	1		1	1
	Unemployed	1.0 (0.6–1.6)		3.0 (1.7–5.4) **	2.0 (1.1–3.8) *
Engaged in Physical Exercise	Yes	1	1	1	1
	No	3.9 (1.9–7.9) **	2.8 (1.3–5.9) **	1.9 (1.1–3.3) *	1.4 (0.7–2.9)
Cigarette Smoker	Yes	1.1 (0.5–2.2)		0.9 (0.5–2.1)	
	No	1		1	
Alcohol Drinking Habit	Yes	1.4 (0.8–2.3)		1.6 (0.9–2.9)	
	No	1		1	
Family History of Diabetes	Yes	1		1	
	No	0.9 (0.5–1.4)		1.2 (0.7–2.0)	
Comorbidities	Yes	0.7 (0.4–1.1)		0.8 (0.5–1.4)	
	No	1		1	
Family Support	Yes	1	1	1	
	No	2.3 (1.3–4.3) **	1.5 (0.8–2.9)	1.2 (0.8–3.2)	
T2DM related complication	Yes	1.1 (0.6–1.9)		1.3 (0.8–2.3)	
	No	1		1	
Check blood glucose	Regularly check	1		1	
	Don’t check	0.9 (0.4–2.3)		1.3 (0.6–3.1)	
Access to nutritional education	Yes	1		1	
	No	1.3 (0.8–2.1)		1.4 (0.8–2.5)	
Access to information	Yes	1		1	1
	No	1.4 (0.8–2.4)		3.6 (2.0–6.3) **	2.8 (1.5–5.4) **
Disease Duration	< 5 years	1		1	
	> = 5 years	1.5 (0.9–2.4)		1.4 (0.8–2.4)	
Glycemic Control	Controlled	1		1	
	Poorly controlled	1.0 (0.6–1.7)		1.6 (0.9–2.9)	
Perceived barrier to Dietary adherence	Yes	0.5 (0.2–0.9) *	0.5 (0.2–1.1)	(0.6–1.9)	
	No	1	1	1	
T2DM related Knowledge	Adequate	1	1	1	1
	Inadequate	2.4 (1.5–3.9) **	1.8 (1.1–2.9) *	2.2 (1.2–3.9) **	1.7 (1.1–3.2)*

Note. Each adjusted odds ratio is adjusted for the remaining variables shown in the Table. AOR = adjusted odds ratio; COR = crude odds ratio.

*P < 0.05

**P < 0.01.

## Discussion

According to Sami et al. [13], adherence to dietary counselling and effective dietary practice positively affect managing diabetes and preventing its complication. This study reported important information about adherence to dietary counselling and associated factors among patients with T2DM attending the diabetes clinic at public hospitals in Addis Ababa, Ethiopia.

The present study’s reported nonadherence to dietary counselling, 67.3%, was comparable with other previous studies conducted in Northern Ethiopia (64.1%) [18] and Lesotho (66.1%) [40]. The research conducted in Ethiopia (46.8%), Central Ethiopia (55.7%) [19, 41], Botswana (37%) [23], Pakistan (25%) [42], and India (24%) [43] reported lower nonadherence to dietary counselling as compared to the present study. On the other hand, higher nonadherence levels were reported in Northwest Ethiopia (74.3%) [44] and Yemen (79.0%) [45] compared to the present study. The observed discrepancy might be due to variations in sociodemographic status, geographical location, study setting, and analysis of measurement tools. The variation in nonadherence rates reported in Ethiopia could be due to differences in geographical location, study setting, and counselling services.

Nonadherence to dietary counselling could lead to poorly controlled blood glucose levels and cause diabetes-related complications among patients with T2DM [13, 46]. In this study, patients who developed complications reported high nonadherence to dietary counselling than patients who didn’t develop complications. However, the association between T2DM related complications and dietary nonadherence was not significant. Further longitudinal research is needed to understand how nonadherence leads to complications.

Patients with T2DM are expected to maintain a healthy diet, high protein, ample vegetables and fruits, low glycemic index foods, and low saturated fat [47, 48]. A healthy diet will help patients with T2DM to control their blood sugar levels and prevent consequent complications related to the disease [46]. However, keeping these healthy diet recommendations will be challenging for food-insecure patients with T2DM. Pieces of evidence show that food-insecure patients with diabetes consume fewer fruits, vegetables, proteins, and it is challenging to eat regularly and follow diet plans [26, 49]. In this study, more than half of the respondents were food insecure. This result was higher than the study conducted in Kenya [50].

Conversely, a higher prevalence of food insecurity (85%) was reported in Iran among patients with diabetes [51]. Moreover, food-insecure patients with T2DM were more likely to be nonadherent to dietary counselling than food-secure counterparts. The study result by Berkowitz and his colleagues [52] supported this; food insecurity has negatively affected overall dietary quality consumption, which was related to poor glycemic control.

In the stratum-specific analysis, information access and diabetes knowledge were significantly associated with nonadherence to dietary counselling among food-insecure patients. Improving adherence to dietary counselling in patients with T2DM necessitated screening for food insecurity [53, 54] and diabetes dietary education tailored to food-insecure patients taking their income and food availability into account [20]. Increasing employment opportunities [20], improving food access through subsidization [55], and ensuring economic self-sufficiency [56] could be important in ensuring food security and dietary adherence among patients with T2DM. Programs can specifically reduce the financial burden of food-insecure groups by providing vouchers for key foods such as fruits and vegetables, carbohydrate-containing foods with a low glycemic index, and omega-3 oils, subsidizing fruits and vegetables and ensuring their affordability [20, 57].

In this study, only 25.9% of patients with T2DM were not engaged in physical exercise. This result is comparable with studies conducted in southwest Ethiopia [38], Nepal [43], and Botswana [23] and higher than the study conducted in Iran [58]. In our study, patients who did not engage in physical exercises reported significantly higher nonadherence to dietary counselling than patients who engaged in physical exercises (p<0.01). A significant association between physical activity and adherence to dietary counselling is supported by similar other studies [59, 60]. This result may imply patients who regularly exercise may focus more on their diet plan and eating pattern than those who did not engage in physical exercises. However, the stratum-specific analysis revealed that the significant association between physical exercise and dietary adherence was only identified among food secure group.

Access to information can encourage and improve diabetes patients’ attention to follow dietary advice, and promising results have been reported [39]. In our study, access to information related to diabetes nutrition was significantly associated with nonadherence to dietary counselling. This finding suggests that increasing access to information could be a key factor in improving adherence to dietary advice. Distributing freely available written information, such as booklets, brochures, or leaflets, at public health institutions and supporting nutrition education programs through public mass media could be beneficial, particularly for food-insecure groups.

Behavioral and cognitive representations of diabetes and diabetes knowledge are vital for improving diabetes self-management practices and adherence to dietary recommendations. Patients with T2DM need to be knowledgeable to follow dietary guidelines and improve glycemic control [39]. In our study, 58.5% of patients with T2DM had inadequate diabetes-related knowledge. This result was comparable with the studies conducted in North Ethiopia [61], Bangladesh [62], Zimbabwe [63], and Congo [64]. The level of inadequate knowledge was lower than the studies conducted in Southwest Ethiopia [65]. The variation could be attributed to differences in measurement scale analysis, study setting, and sociodemographic characteristics.

This study revealed that nonadherence to dietary counselling was significantly associated with a patient’s diabetes knowledge score. This finding was consistent with two similar studies [66, 67]. Inadequate diabetes knowledge influenced food choice and dietary patterns [68]. Implementing innovative and tailored diabetes education could improve diabetes knowledge and dietary practice. However, education and diabetes knowledge alone are not adequate to bring change in practice; a comprehensive understanding of the factors contributing to dietary Counselling adherence is mandatory [69].

The study had limitations that should be recognized. The research scope was limited to patients with T2DM and could be generalized only to patients with T2DM who had regular medical check-ups in public hospitals in Addis Ababa. The assessment made on dietary counselling and physical exercise was self-reported. We could not establish a temporal relationship between food security and other factors and adherence to dietary counselling. We did not make a qualitative inquiry to identify possible reasons for low adherence to dietary counselling among patients with T2DM. Thus, detailed longitudinal studies are required to investigate the relationship between nonadherence to dietary counselling and the food security of patients with T2DM. Another limitation is the lack of a specific tool to measure “Access to information.” The participants’ access to information was measured by asking where they got their diabetes information. Additional qualitative studies could be essential to identify barriers to dietary Counselling adherence.

## Conclusions

More than two-thirds of patients with T2DM were nonadherent to dietary counselling, and the level appears to be high. More than half of the respondents were food insecure. Physical activity level, diabetes knowledge, food security, and access to information related to diabetes nutrition were identified as significant determinants of poor adherence. This has significant implications for improving diabetes education with increased hospital information access. The presence of national dietary guidelines, as well as improved food security, could be critical. Further actions should be taken to improve dietary practice by considering the differences in contributing factors between food-secure and food-insecure patients.

## Supporting information

S1 Table Dietary counselling adherence distribution of patients with T2DM at selected public hospitals, Addis Ababa, 2019. (DOCX)

S2 Table Coefficients of multiple regression including interaction terms. (DOCX)
