# Supplementary material for: Food security status of patients with type 2 diabetes and their adherence to dietary counselling from selected hospitals in Addis Ababa, Ethiopia: A cross-sectional study
Source: PLoS One. 2022 Apr 14;17(4):e0265523. doi: 10.1371/journal.pone.0265523 (PMC9009691; doi:10.1371/journal.pone.0265523)
Supplement: S1 Table — (DOCX) [file pone.0265523.s001.docx]

**S1 Table. Dietary counseling adherence distribution of patients with T2DM at selected public hospitals, Addis Ababa, 2019**

| **PDAQ Items** | **PDAQ Score (days/week)** | | | | | | | |
| --- | --- | --- | --- | --- | --- | --- | --- | --- |
|  | **0 (%)** | **1 (%)** | **2 (%)** | **3 (%)** | **4 (%)** | **5 (%)** | **6 (%)** | **7 (%)** |
| Follow Healthful eating plan | 34 (5.6% | 61 (10.1%) | 125 (20.8%) | 51 (8.5%) | 68 (11.3%) | 55 (9.1%) | 30 (5%) | 178 (29.6%) |
| Consume Fruit and vegetable | 61 (10.1%) | 126 (20.9% | 168 (27.9%) | 98 (16.3%) | 63 (10.5%) | 15 (2.5%) | 8 (1.3%) | 63 (10.5%) |
| Consume Carbohydrate-containing foods with a low Glycemic Index | 88 (14.6%) | 171 (28.4%) | 172 (28.6%) | 74 (12.3%) | 30 (5%) | 30 (5%) | 13 (2.2%) | 24 (4%) |
| Consume Foods high in sugar | 330 (54.8%) | 157 (26.1%) | 70 (11.6%) | 23 (3.8%) | 11 (1.8%) | 7 (1.2%) | 2 (0.3%) | 2 (0.3%) |
| Consume Foods high in fiber | 177 (29.4%) | 189 (31.4%) | 103 (17.1%) | 56 (9.3%) | 46 (7.6%) | 21 (3.5%) | 5 (0.8%) | 5 (0.8%) |
| Spaced carbohydrates evenly throughout the day | 226 (37.5%) | 167 (27.7%) | 141 (23.4%) | 35 (5.8%) | 22 (3.7%) | 7 (1.2%) | 0 (0%) | 4 (0.7%) |
| Consume Foods that contained omega-3 oils | 276 (45.8%) | 162 (26.9%) | 116 (19.3%) | 20 (3.3%) | 11 (1.8%) | 11 (1.8%) | 2 (0.3%) | 4 (0.7%) |
| Consume Foods high in fat | 200 (33.2%) | 127 (21.1%) | 141 (23.4%) | 77 (12.8%) | 40 (6.6%) | 6 (1%) | 8 (3%) | 3 (0.5%) |
