# Supplementary material for: Food security status of patients with type 2 diabetes and their adherence to dietary counselling from selected hospitals in Addis Ababa, Ethiopia: A cross-sectional study
Source: PLoS One. 2022 Apr 14;17(4):e0265523. doi: 10.1371/journal.pone.0265523 (PMC9009691; doi:10.1371/journal.pone.0265523)
Supplement: S2 Table — (DOCX) [file pone.0265523.s002.docx]

| **Variables** | **Beta** | **95%CI** | **p-value** |
| --- | --- | --- | --- |
| Information access by Food-insecure | 1.69 | 2.6 – 0.75 | <0.001* |
| DM Knowledge by Food-insecure | -0.09 | 0.18 – -0.01 | 0.829 |
| Physical exercise by Food-insecure | -1.43 | -0.37 ­­– -2.49 | 0.008* |

**S2 Table. Coefficients of multiple regression including interaction terms**
